# Differential impact on motility and biofilm dispersal of closely related phosphodiesterases in *Pseudomonas aeruginosa*

**DOI:** 10.1038/s41598-020-63008-5

**Published:** 2020-04-10

**Authors:** Yu-ming Cai, Andrew Hutchin, Jack Craddock, Martin A. Walsh, Jeremy S. Webb, Ivo Tews

**Affiliations:** 1 National Biofilms Innovation Centre, University of Southampton, Southampton, SO17 1BJ UK; 2 Biological Sciences, Institute for Life Sciences, University of Southampton, Southampton, SO17 1BJ UK; 3 Diamond Light Source, Harwell Science and Innovation Campus, Didcot, Oxfordshire OX11 0DE UK; 4 Research Complex at Harwell, Harwell Science and Innovation Campus, Didcot, Oxfordshire OX11 0FA UK; 5 Present Address: Structure and Function of Biological Membranes Lab, Université Libre de Bruxelles, Boulevard du Triomphe, 1050 Bruxelles, Belgium

**Keywords:** Microbiology, Biofilms, Microbial communities, Mutation

## Abstract

In *Pseudomonas aeruginosa*, the transition between planktonic and biofilm lifestyles is modulated by the intracellular secondary messenger cyclic dimeric-GMP (c-di-GMP) in response to environmental conditions. Here, we used gene deletions to investigate how the environmental stimulus nitric oxide (NO) is linked to biofilm dispersal, focusing on biofilm dispersal phenotype from proteins containing putative c-di-GMP turnover and Per-Arnt-Sim (PAS) sensory domains. We document opposed physiological roles for the genes Δ*rbdA* and Δ*pa2072* that encode proteins with identical domain structure: while Δ*rbdA* showed elevated c-di-GMP levels, restricted motility and promoted biofilm formation, c-di-GMP levels were decreased in Δ*pa2072*, and biofilm formation was inhibited, compared to wild type. A second pair of genes, Δ*fimX* and Δ*dipA*, were selected on the basis of predicted impaired c-di-GMP turnover function: Δ*fimX* showed increased, Δ*dipA* decreased NO induced biofilm dispersal, and the genes effected different types of motility, with reduced twitching for Δ*fimX* and reduced swimming for Δ*dipA*. For all four deletion mutants we find that NO-induced biomass reduction correlates with increased NO-driven swarming, underlining a significant role for this motility in biofilm dispersal. Hence *P. aeruginosa* is able to differentiate c-di-GMP output using structurally highly related proteins that can contain degenerate c-di-GMP turnover domains.

## Introduction

*Pseudomonas aeruginosa* is a gram-negative bacterium known for its environmental versatility. As an opportunistic pathogen, *P. aeruginosa* causes disease, particularly in immune compromised individuals, and is a major source of morbidity and mortality in cystic fibrosis (CF) patients with chronic colonisation in lungs and airways^1^. The ability of *P. aeruginosa* to form biofilms within CF patients elicits increased antibiotic tolerance, which makes treatment of infections problematic in clinical settings^2^.

*P. aeruginosa* biofilm formation and dispersal are known to correlate with intracellular concentrations of the secondary messenger, cyclic dimeric-GMP (c-di-GMP)^3,4^. The production and degradation of c-di-GMP relies on two enzymatic activities. Diguanylate cyclases (DGCs) synthesise c-di-GMP from two GTP molecules, while phosphodiesterases (PDEs) hydrolyse the secondary messenger to linear pGpG^3^. *P. aeruginosa* PAO1 encodes 17 different proteins with a DGC domain, 8 with a PDE domain, and 16 that contain both of these domains, with the DGC N-terminal to the PDE domain^5^.

The transition between planktonic and biofilm lifestyles is accompanied by extracellular polymeric substance (EPS) production and motility changes. Extensive studies have characterised the link between flagella, pili and biofilm morphologies^6–8^. The main motility types in *P. aeruginosa* PAO1 are flagella mediated swimming and pili mediated twitching. Further, the complex swarming motility is required for dispersal, which relies on flagella, pili and surfactants, and involves multicellular group movement on a surface^9,10^. The regulatory relationships between c-di-GMP and different motility types have been investigated for a number of *P. aeruginosa* proteins. Swimming motility in PAO1 is known to be reduced through deletion of the *rbdA* gene, which encodes for a DGC-PDE^11^, or over-expression of the DGC SadC^12^, while the polarly localised PDE DipA is necessary for c-di-GMP heterogeneity that reduces flagellar velocity and reversals^13^. Further, the DGC SadC and the PDE BifA are involved in the regulation of swarming motility^14,15^, required for formation and dispersal of biofilms^16–19^. Swarming behaviour in *P. aeruginosa* PA14 has been linked to intracellular c-di-GMP concentration through the flagellar stators MotAB and MotCD^20^. The two DGCs, SadC and RoeA both promote biofilm formation, but individually control flagellar motility or the production of EPS, respectively^21^. These examples illustrate how multiple proteins have discrete phenotypic outputs to adjust the intracellular c-di-GMP and regulate biofilm formation and dispersal.

Within *P. aeruginosa*, intracellular c-di-GMP levels can be regulated by a number of environmental cues, including nutrient availability and the presence of nitric oxide^22,23^. Indeed, DGC and PDE-containing proteins typically also have one or several putative regulatory domains. An example of this is provided by the PAS (Per-Arnt-Sim) domain, which is a ubiquitous regulatory domain known to control dimerisation^24^. In *P. aeruginosa* PAO1, twelve genes code for proteins with PAS domains linked to DGC domains, nine of which additionally contain PDE domains^5^. The recurrence of proteins with similar architecture which may play a role in biofilm regulation poses a key question: If DGCs and PDEs regulate the transition from sessility to the planktonic state, do these proteins provide an element of redundancy within the cell or do they coordinate individual behaviours that contribute to this phenotypic change? To address this question, data on how different DGC/PDEs diversify in regulating biofilm dispersal are urgently needed.

Using deletion mutants we present data on c-di-GMP levels, motility, EPS production, biofilm structure and nitric oxide induced dispersal for four PAS domain containing DGC-PDE proteins. Reduced NO-induced biofilm dispersal was observed in Δ*rbdA*, Δ*pa2072* and Δ*dipA* biofilms, while Δ*fimX* showed increased dispersal upon exposure to nitric oxide. The NO-induced biomass reduction correlates with an increase of NO-driven swarming, suggesting a significant role for this motility in biofilm dispersal. This study is focussed on two pairs of proteins: (1) the RbdA/PA2072 pair shows how proteins with similar protein structures can have very different function in c-di-GMP regulation, biofilm formation and motility; (2) the FimX/DipA pair shows how proteins containing degenerate, pseudo-enzymatic domains are essential for motility and biofilm structure. This study highlights how *P. aeruginosa* is able to differentiate signals from multiple c-di-GMP outputs in order to regulate complex biological processes associated with biofilm development.

## Materials and methods

### Bioinformatics and homology modeling

To determine domain architectures, the EMBL SMART web server was used (http://smart.embl-heidelberg.de)^25^. To check for the presence of key catalytic residues, individual DGC and PDE domains were aligned against catalytically active proteins for which a structure had been deposited in the PDB, using the CLUSTAL web server (https://www.ebi.ac.uk/Tools/msa/clustalo/)^26^. Protein structure homology modelling was carried out using the SWISS-MODEL server (https://swissmodel.expasy.org)^27^, using the DGC template structure PleD (PDB 2V0N^28^) and the PDE template MorA (PDB 4RNH^29^).

### Bacterial strains and culture media

Bacterial strains and plasmids used in this study are listed in Table S1. Routine overnight cultures were grown in lysogeny broth (LB) medium. Biofilms were grown in modified M9 minimal medium^22^. Antibiotics were used at the following concentrations as previously described^30^: for *P. aeruginosa* PAO1, gentamicin was used at 60 μg/ml, carbenicillin at 400 μg/ml, kanamycin at 300 μg/ml and tetracycline at 60 μg/ml; for *E. coli* S17-1, gentamicin was used at 15 μg/ml, ampicillin at 100 μg/ml, tetracycline at 30 μg/ml, kanamycin at 50 μg/ml and streptomycin at 50 μg/ml.

### Isogenic *P. aeruginosa* PAO1 mutants

Isogenic mutants were constructed by replacing the coding regions of each gene with a gentamicin resistance cassette as previously described^30^. The Gm cassette was amplified from pPS856^31^ using primers Gm-F and Gm-R (primer sequences listed in Table S2). For each gene, flanking up- and downstream regions (approximately 350–400 bp) were amplified by standard PCR, digested with EcoRI and HindIII, and ligated to the amplified Gm cassette. To generate KO plasmids, the PA-up-Gm-PA-dn fragments were inserted into the SmaI site of the pEX100T suicide vector, which contains the *sacB* gene for counter selection and an ampicillin resistance gene on the backbone. Plasmids were introduced into *E. coli* S17-1 by chemical transformation and then transferred into PAO1 by conjugation. Transconjugants were first selected on *Pseudomonas* isolation agar containing 60 μg/ml Gm and then patched onto LB agar with 10% sucrose and 60 μg/ml Gm and LB agar with 400 μg/ml carbenicillin. Colonies that only grew on LB agar with 10% sucrose and 60 μg/ml Gm but not on LB agar with 400 μg/ml carbenicillin were selected as double-recombinant mutants and confirmed by PCR and sequencing. For the generation of the double Δ*pilA*Δ*fliM* mutant, the gentamycin cassette was used for the introduction of a single mutant, with a kanamycin cassette used to introduce the second mutation. This Km cassette was amplified from pCR4-TOPO (Invitrogen) using primers Km-F and Km-R.

### Batch culture *P. aeruginosa* PAO1 biofilms

Overnight cultures were diluted into fresh M9 media (OD_600nm_ ~0.01) to inoculate microtiter plates (used for initial biofilm dispersal screening) or MatTek plates (P35G-1.5-14-C, used for confocal microscopic biofilm studies), using 100 μl or 3 ml of diluted culture, respectively. Microtiter plates were incubated statically, while MatTek plates were shaken at 50 rpm to create shear force facilitating biofilm formation. M9 media in each plate was changed every 24 hrs. Biofilms in microtiter plates were stained with 0.1% (w/v) crystal violet and dissolved in 30% (v/v) acetic acid. Biofilms in MatTek plates were stained with LIVE/DEAD BacLight (Invitrogen) and examined by confocal laser scanning microscopy. Crystal violet staining was quantified at a wavelength of 584 nm, while a wavelength of 488 nm was used for SYTO-9 and 561 nm was used for propidium iodide excitation. At least 3 image stacks were taken from random locations in each MatTek plate. Biofilms were analysed by COMSTAT^32^ and ImageJ. For NO donor experiments, 250 μM Spermine NONOate (S150, Sigma-Aldrich) was added to the microtiter or MatTek plates and then incubated at 37 °C for 2 hrs to trigger dispersal.

### Determination of the relative level of c-di-GMP *in vivo* (adapted from Rybtke *et al*.)

The c-di-GMP reporter plasmid (courtesy of A. Filloux, Imperial College London, UK) encodes green fluorescent protein under the control of a c-di-GMP responsive *cdrA* promoter and allows quantification of relative c-di-GMP levels^33^. This plasmid was introduced into *P. aeruginosa* by conjugation. Cultures were inoculated in 10 ml M9 with 60 μg/ml tetracycline (OD_600nm_~0.001) and incubated for 22 hrs at 37 °C by shaking at 180 rpm. Strains without reporter were used as negative controls. For NO donor treatment, 25 μM S150 was added to cultures before incubation for an additional 2 hrs. Bacterial cultures (100 μl) were transferred into black polystyrene flat bottom Greiner CELLSTAR 96-well plates to determine fluorescence intensity, and into clear polystyrene flat bottom CELLSTAR 96-well plates to determine cell density. Arbitrary fluorescence intensity units (FIU) were determined using a 485 nm sharp-cut excitation filter and a 520 nm sharp-cut emission filter with a gain of 1500 on a BMG LABTECH FLUOSTAR plate reader. Relative fluorescence units (RFU) were determined from the FIU and normalised against cell density (OD)^34^:

$$(\frac{GFPreporter-GFPmedium}{ODreporter-ODmedium})-(\frac{GFPwt-GFPmedium}{ODwt-ODmedium})$$

### EPS extraction and quantification

Biofilms with an initial inoculum of diluted overnight culture (OD_600nm_ ~0.01) were cultured in tissue-culture treated dishes (Corning, UK, D × H 100 mm × 20 mm). Cell scrapers were used to harvest biofilms attached to the bottom of each dish. Bacteria were re-suspended in 3 ml PBS, vortexed, and 100 μl were taken for CFU counts before centrifugation at 18514 *× g* for 10 mins. Supernatants were directly analysed for soluble polysaccharide and protein content, while centrifugation pellets were resuspended in 2 ml 0.85% NaCl and 12 μl 37% formaldehyde (Sigma-Aldrich, UK). The resuspended samples were vortexed, incubated at 4 °C for 1 hr before addition of 2.5 ml 0.8 M NaOH, further incubation for 3.5 hrs and centrifugation at 4000×*g* (40 mins, 4 °C). These supernatants were then freeze-dried and redissolved in water adjusted to pH 7 using H_2_SO_4_. The polysaccharide content of each sample was determined using the phenol-H_2_SO_4_ method^35^, quantifying absorbance at 492 nm with glucose used as a standard. The protein content was determined using the Coomassie (Bradford) protein assay kit (Thermo Scientific, UK), quantifying absorbance at 595 nm using bovine serum albumin (Thermo Scientific, UK) as a standard.

### Determination of swarming motility

Swarming assay was adapted from Rashid MH and Kornberg A^36^. Briefly, swarming agar plates were prepared from 0.5% (w/v) agar (Sigma Aldrich, UK) in 8 g/L nutrient broth (Oxoid, UK) and 5 g/L glucose (Sigma Aldrich, UK). NO donor swarming plates additionally contained SNP at a final concentration of 1 μM. Swarming plates were dried under laminar flow for 40 mins and inoculated with 3 μl late exponential culture. Plates were incubated at 37 °C for 24 hrs under normal laboratory light conditions. For swarming agar with SNP, 10 mM SNP stock solution was made in sterilised PBS and then incorporated into 50 °C swarming agar to a final concentration of 1 μM.

### Determination of swimming motility

Swimming assay was adapted from Rashid MH and Kornberg A^36^. Briefly, tryptone broth (10 g/L tryptone, Oxoid, UK) with 5 g/L NaCl and 0.3% (w/v) agarose (Melford, UK) was used to prepare agar plates for swimming motility assays. The plates were dried under laminar flow for 15 mins and inoculated from an overnight LB agar plate with a sterile 2 μl pipette tip. Incubation at 30 °C was carried out for 20 hrs.

### Determination of twitching motility

Twitching assay was adapted from Rashid MH and Kornberg A^36^. Briefly, twitching agar plates were prepared from LB broth (Miller, UK) with 1% (w/v) agar (Sigma Aldrich, UK). Plates were dried under laminar flow for 1 hr and inoculated from an overnight LB agar plate by stabbing with a sterile toothpick through the agar to the bottom of the plate. Plates were incubated at 37 °C for 24 hrs.

### Statistical analyses

Biofilm formation assays in microtiter plates and intracellular c-di-GMP assays were assessed using the two-tailed Student’s T-test. Confocal microscopic biofilm analysis, EPS production and motility assays were assessed using the two-tailed Welch T-test, without the assumption of an equal standard deviation. Statistical significances were P < 0.001 for all measurements reported unless otherwise stated. Statistics and graphs were produced using GraphPad Prism.

## Results

### The *P. aeruginosa* genome encodes twelve PAS-DGC-PDE proteins

The *P. aeruginosa* PAO1 genome encodes 41 proteins with either diguanylate cyclase (DGC) or phosphodiesterase (PDE) domains^5,37^. These are often found in multi-domain proteins with a variety of auxiliary domains, such as putative regulatory domains or membrane segments. The additional domains are almost exclusively found N-terminal to the enzymatic DGC or PDE domains^5^. Most common are transmembrane helices, while the PAS domain is the second most commonly found domain. PAS domains, named after the Per-Arnt-Sim homology^24^, are widely used as regulatory domains in a number of organisms. To better understand the widespread usage of this architecture in DGC and PDE proteins, we studied the multi-domain proteins that contain PAS sensor domains together with DGC and/or PDE domains.

**Figure 1:**
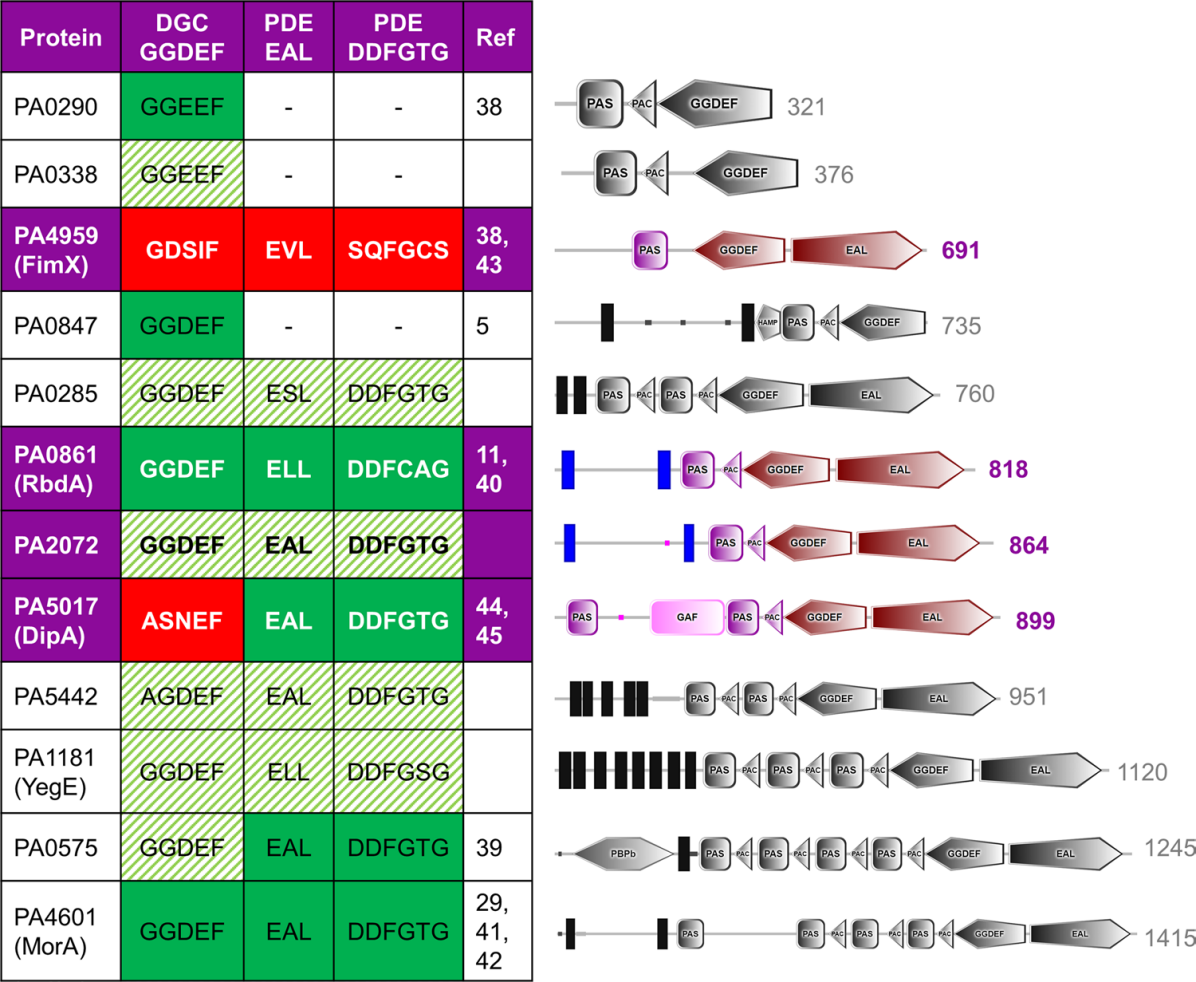
*P. aeruginosa* proteins with PAS and DGC/PDE domains. For each protein, the domain structure is shown on the right as determined by the SMART domain prediction server^25^, with the sequence length also indicated. The observed sequence of the signature motifs for DGC domains (GGDEF) and PDE domains (EAL, DDFGTG) are given. Where enzymatic activity has been determined experimentally, this is indicated in green (or red, for no activity). Sequence alignment and homology modelling predicts the remaining proteins to be enzymatically active, as indicated by shading in green. NB. The N-terminal REC domain in FimX (residues 1-120) was not assigned by SMART^25^ and is not shown here.

Twelve proteins were identified in *P. aeruginosa* PAO1 that contained PAS domains together with DGC and/or PDE domains. These proteins are listed in Fig. 1 and are named *pa0285*, *pa0290*^38^, *pa0338*, *pa0575*^39^, *pa0847*^5^, *pa0861* (*rbdA*^11,40^), *pa1181* (*yegE*), *pa2072*, *pa4601* (*morA*^29,41,42^), *pa4959* (*fimX*^38,43^), *pa5017* (*dipA*^44,45^) and *pa5442*. Of these, eight are associated to the membrane, as evidenced by the presence of at least one predicted transmembrane segment. When analysed through the SMART domain prediction server, the number of predicted PAS domains in these proteins varies from one to four. Several of the twelve proteins contain further sensory or regulatory domains, for example PA0575 also contains a periplasmic substrate-binding domain commonly associated with bacterial ABC transporters^46^, PA5017 (DipA) carries an additional sensory GAF domain^47^, PA0847 contains a transmembrane signal-mediating HAMP domain^48^ and PA4959 (FimX) contains a phosphoaccepting REC domain^49^.

All twelve proteins contain DGC domains, while only nine of these contain an additional PDE domain. We have investigated conservation of the enzymatic signature motifs (G/A)G(**D/E)E**F and **E(**A/S/L)L as an indication of catalytic competence. The GG**DE**F motif of DGC domains contains the conserved aspartate and glutamate residues required for catalysis. The **E**AL and **DD**FGTG motifs are both required for catalytic activity of PDE domains, as they contribute the catalytic glutamate and two metal coordinating aspartates to the catalytic centre, respectively. Homology modelling with SWISS-MODEL was used as a second validation step. DGC domains were modelled after the substrate-bound structure of *Caulobacter crescentus* PleD (PDB 2V0N^28^), and PDE domains (specifically EAL domains) were modelled after the substrate-bound structure of *P. aeruginosa* MorA (PDB 4RNH^29^).

Ten of the twelve PAS-DGC and PAS-DGC-PDE domain proteins have been experimentally shown to be active, or they show conservation of catalytic residues. PA4959 (FimX) and PA5017 (DipA) have been experimentally determined to have degenerate, inactive DGC domains^44,50^. While the PDE domain of DipA has been shown to be active, PDE activity of FimX remains a somewhat contested subject due to conflicting reports of c-di-GMP hydrolysis^38,43,44,50–52^. The degenerate catalytic EAL and DDFGTG motifs and homology modelling indicate that metal binding should be compromised in FimX due to the lack of metal coordinating side chains. Therefore, FimX is listed here as an inactive pseudo-enzyme. In Fig. 1, solid green shading indicates enzyme domains previoulsy reported to be active, with red solid shading denoting enzymatic domains previously reported as inactive; our predictions of enzymatic activity from both alignment and homology modelling are shown in striped green shading.

### Identification of NO response PAS-DGC-PDE proteins

**Figure 2:**
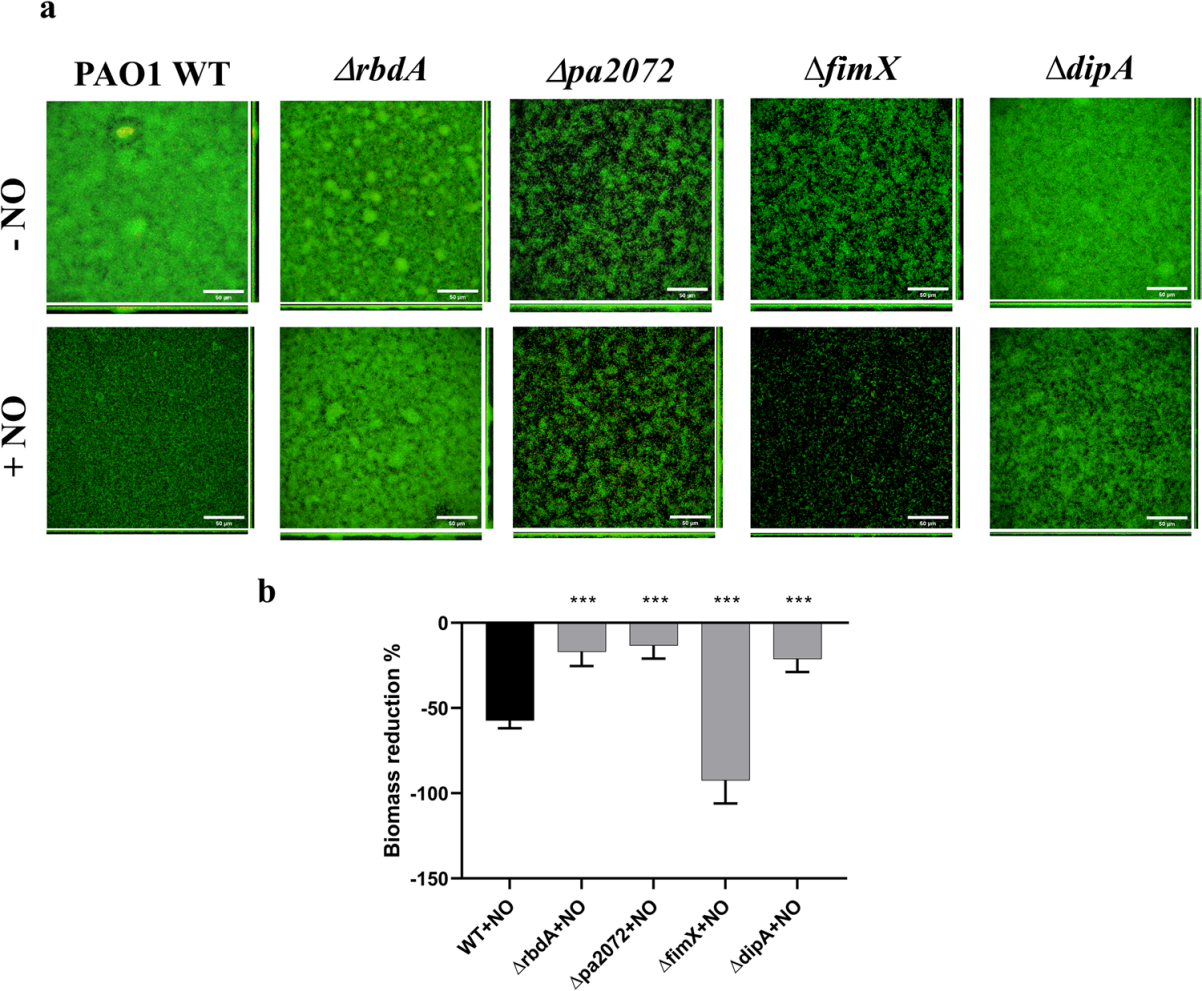
NO-induced biofilm dispersal. **(A)** Confocal laser scanning microscopy images used for phenotypic analysis of 72 hrs mature biofilms before and after 2 hrs treatment with 250 μM S150 for PAO1 WT and the deletion mutants Δ*rbdA*, Δ*pa2072*, Δ*fimX* and Δ*dipA* (scale bar 50 μm). **(B)** COMSTAT analysis of biomass reduction after S150 treatment, as normalised against WT, highlighting differences in biomass reduction for the gene deletion mutants. The Welch’s T-test is used to determine significances, where ***denotes a confidence level of p < 0.01. Data acquired from 3 independent experiments.

To determine which of the twelve PAS domain containing DGC/PDE proteins responded to stimulation with NO, we studied biofilm formation and NO-induced dispersal by individually deleting each gene using homologous recombination. Biofilms were cultured for 72 hrs and screened for NO-induced dispersal using a microtiter plate assay and crystal violet staining. Among the 12 deletion mutants, two pairs of proteins were selected. RbdA and PA2072 are two proteins with similar structure, and Δ*rbdA* and Δ*pa2072* display prominent phenotypes, as both showed impaired responses to NO induced dispersal when compared to wild type (WT). The two pseudo-enzymes DipA and FimX were also selected, as *ΔfimX* displayed an increased dispersal and *ΔdipA* showed a reduction in dispersal in comparison to WT. Confocal laser scanning microscopy and COMSTAT analysis of these deletion mutants are shown in Fig. 2a,b, respectively. While WT biofilm biomass reduced 57 ± 5% after NO treatment, Δ*rbdA*, Δ*pa2072* and Δ*dipA* showed 17 ± 8%, 13 ± 8% and 21 ± 8% reduction. Conversely, Δ*fimX* biofilms dispersed more than 92 ± 14%.

**Figure 3:**
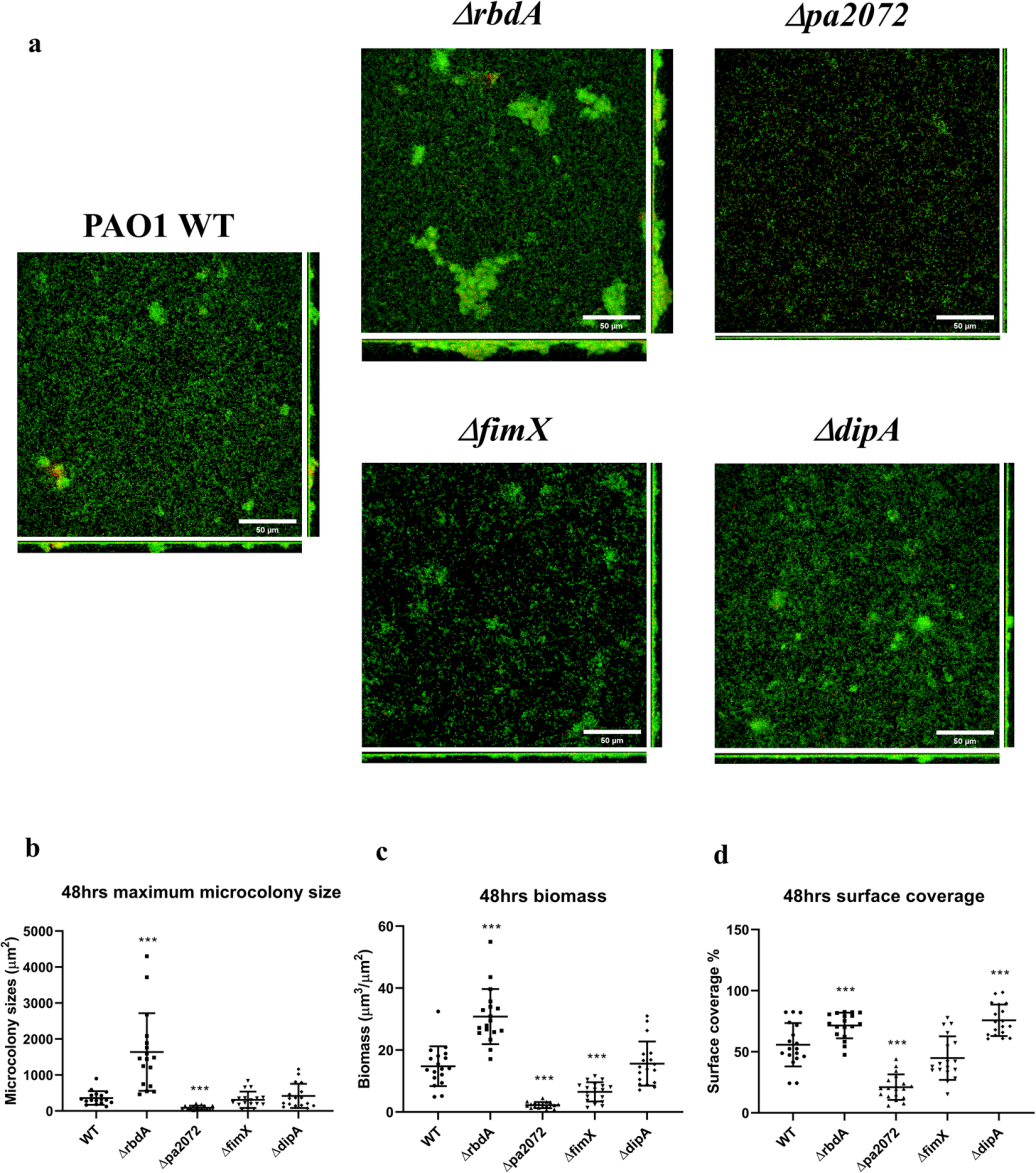
Biofilm morphologies of the four mutants of interest. Biofilms formed after 48 hrs for Δ*rbdA*, Δ*pa2072*, Δ*fimX*, and Δ*dipA*, compared with PAO1 WT. **(A)** Confocal laser scanning micrographs at 63× magnification (scale bar 50 μm); live cells are stained with SYTO-9 (green); dead cells are stained with propidium iodide (red). **(B)** Quantification of microcolony size. **(C)** Quantification of biofilm biomass. **(D)** Quantification of surface coverage. For data shown in B-D, the Welch’s T-test was used to determine significances, where ***denotes a confidence level of p < 0.01. Data acquired from 3 independent experiments.

To highlight differences in biofilm formation, early stage biofilms (cultured for only 48 hrs) were investigated. Δ*rbdA* formed thicker biofilms than WT PAO1 (Fig. 3a), with significantly increased microcolony size (5.6 ± 3.2-fold, P < 0.001), biomass (2.1 ± 0.6-fold, P < 0.001) and surface coverage (1.3 ± 0.5-fold, P < 0.01) (Fig. 3b–d). In contrast, Δ*pa2072* showed an opposite biofilm phenotype with reduced biomass (6.6 ± 0.6-fold) and surface coverage (2.7 ± 2.2-fold), as well as smaller microcolonies (4.5 ± 1-fold) (Fig. 3b–d). The data suggests that PA2072 and RbdA play opposite roles in PAO1 biofilm formation, which is surprising given their very similar domain architectures.

On the other hand, Δ*fimX* biofilms showed 56 ± 8% less biomass than WT, Fig. 3c, but an unchanged maximum microcolony size and surface coverage (Fig. 3b,d). The different biofilm structure might be rooted in less compact microcolonies as seen in Fig. 3a, and this is even more pronounced in mature biofilms (Fig. 2). Δ*dipA* showed a slightly increased surface coverage (1.4 ± 0.4-fold, P < 0.01) (Fig. 3d), but otherwise comparable biomass and microcolony sizes to WT (Fig. 3b,c).

### The correlation of intracellular c-di-GMP concentration and biofilm formation breaks down for pseudo-enzymes FimX and DipA

**Figure 4:**
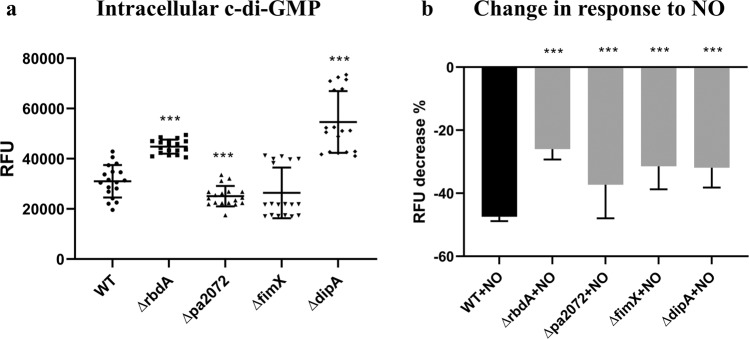
Levels of c-di-GMP in planktonic culture, as quantified using a GFP reporter. **(A)** Relative fluorescence units, which are proportional to intracellular c-di-GMP concentration, normalised to PAO1 WT. **(B)** The NO-induced decrease in relative fluorescence, and thus relative c-di-GMP concentration, given in percentages. The Student’s T-test was used to determine significances, where ***denotes a confidence level of p < 0.01. Data acquired from 3 independent experiments.

Biofilm formation and dispersal are closely linked to c-di-GMP, and the link between reduced intracellular c-di-GMP and NO triggered dispersal is well known^22^. To determine the relative intracellular c-di-GMP concentration we used a c-di-GMP responsive GFP reporter before and after NO treatment. As shown in Fig. 4a, intracellular c-di-GMP levels were significantly increased for Δ*rbdA* (1.5 ± 0.2-fold, P < 0.001) but lowered for Δ*pa2072* (1.2 ± 0.1-fold, P < 0.001), correlating well with respective increased and decreased biofilm formation (Fig. 3). Both mutants showed an impaired c-di-GMP response to NO. While the reduction of intracellular c-di-GMP in WT was 47 ± 1% upon NO challenge, the deletion mutants showed 26 ± 3% (Δ*rbdA*) and 37 ± 5% (Δ*pa2072*) reductions in c-di-GMP levels, respectively (Fig. 4b).

This relationship does not hold for the pseudo-enzymes. Higher than WT c-di-GMP levels were observed for Δ*dipA* (1.8 ± 0.2-fold) Fig. 4a, in spite of biofilm formation properties similar to WT (Fig. 3). In contrast, Δ*fimX* showed a c-di-GMP level comparable to WT (Fig. 4a), despite reduced biofilm formation (Fig. 3). Also surprising is that Δ*fimX* showed increased NO-induced biofilm dispersal, which should normally correlate with c-di-GMP reduction, but in this mutant reduction in c-di-GMP level is less than seen for WT, Fig. 4b. Therefore, biofilm properties are not exclusively dependent on reduction of the overall c-di-GMP concentration.

### EPS and motility phenotypes distinguish Δ*rbdA*, Δ*pa2072*, Δ*dipA and* Δ*fimX*

**Figure 5:**
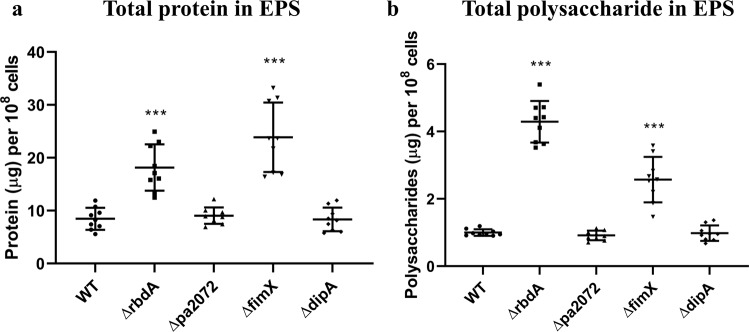
Constituent components of the EPS within the different examined mutants. Quantification of EPS from 10^8^ cells of 48 hrs biofilms of Δ*rbdA*, Δ*fimX*, Δ*dipA* and Δ*pa2072*, compared with PAO1 WT. Total protein **(A)** and polysaccharide **(B)** mass are shown in comparison to PAO1 WT, where the Welch’s T-test was used to determine the significances, and ***denotes a confidence level of p < 0.01. Data acquired from 3 independent experiments.

EPS production and motility are two behavioural traits linked to regulation by DGCs^21^ and PDEs^15,53^, with both known to influence biofilm formation and dispersal^54–57^. The protein and polysaccharide content of the biofilm EPS matrix was therefore determined for each mutant (normalised to cell density). Consistent with the increased biomass and surface coverage (Fig. 3c,d), Δ*rbdA* biofilms displayed increased total protein (2.2 ± 0.1-fold) and polysaccharide (4.3 ± 0.3-fold) compared to WT biofilms (Fig. 5). The structurally related protein Δ*pa2072*, however, produced comparable EPS with WT despite reduced biomass, pointing at further functional differences between the two similar proteins.

Comparable EPS production was observed for *ΔdipA* and WT biofilms, consistent with their similar biomass (Figs. 2 and 3). However, a differentiation is seen for Δ*fimX*, with an increased EPS polysaccharide (2.6 ± 0.5-fold) and protein content (2.8 ± 0.2-fold) despite lower biofilm biomass, compared to WT. Previous studies of a *P. aeruginosa* PA14 *fimX* deletion mutant reported increased eDNA production^58^. As eDNA interacts with Psl to facilitate the formation of a biofilm skeleton in *P. aeruginosa*^59^, it is possible that Δ*fimX* biofilms may therefore differ in structure and composition.

**Figure 6:**
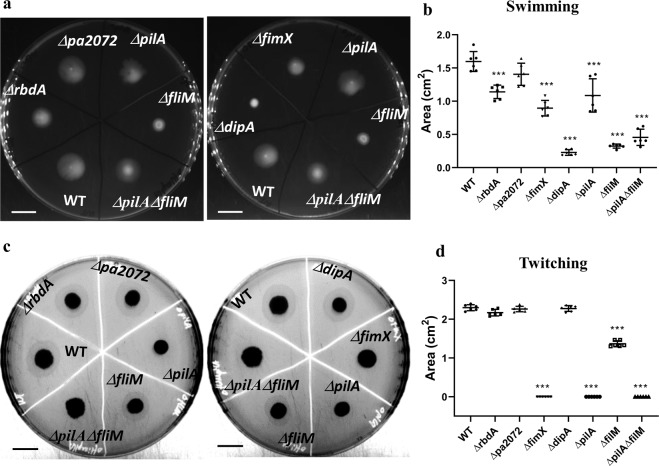
Swimming and twitching phenotypes for the investigated deletion mutants. Swimming agar **(A)** and twitching agar **(C)** for the deletion mutants Δ*rbdA*, Δ*pa2072*, Δ*fimX* and Δ*dipA* compared with PAO1 WT, the flagellum mutant Δ*fliM*, the pili mutant Δ*pilA*, and the pili flagellum double mutant Δ*pilA*Δ*fliM*. Scale bar = 1 cm. Data acquired from 6 independent experiments, shown in **(B**,**D)**, for swimming and twitching areas, respectively, normalised to PAO1 WT. The Welch’s T-test was used to determine significances, where ***denotes a confidence level of p < 0.01.

We went on to characterise bacterial motility of the deletion variants to understand if flagellum or pili play a role in NO induced dispersal. As Δ*rbdA* showed reduced swimming motility (28.5 ± 3% decrease) and the swimming area of Δ*pa2072* was equivalent to WT, further functional differences between these two similar proteins were evident (Fig. 6). Supressed flagellum-mediated swimming motility was observed in Δ*dipA* (86 ± 2% decrease), which is similar to the flagellum mutant Δ*fliM* (80 ± 1% decrease) (Fig. 6a,b). Twitching motility was completely abolished for Δ*fimX*, which is consistent with a previous report^42^ and comparable to the pili mutant Δ*pilA* (Fig. 6c,d).

### NO-induced dispersal correlates with swarming motility

**Figure 7:**
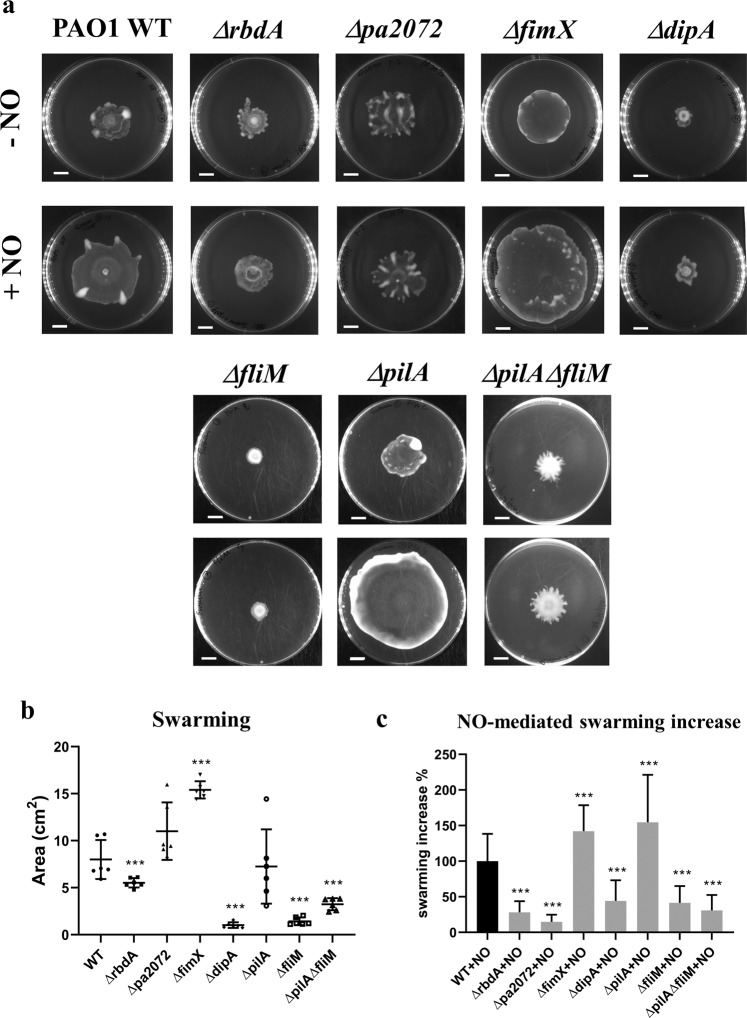
Swarming phenotypes of the examined mutants in the absence and presence of NO. Swarming motilities were determined for the deletion mutants Δ*rbdA*, Δ*pa2072*, Δ*fimX* and Δ*dipA* and compared with PAO1 WT, the flagellum mutant Δ*fliM*, the pili mutant Δ*pilA*, and the pili flagellum double mutant Δ*pilA*Δ*fliM*. (**A**) Swarming agar plates, with scale bar = 1 cm. (**B**) Measured swarming area for each mutant, in the absence of NO, normalised to PAO1 WT. (**C**) The measured increase in swarming areas on NO donor agar (1 μM SNP), normalised to PAO1 WT, compared to swarming areas on agar without an NO donor. The Welch’s T-test was used to determine significances, where ***denotes a confidence level of p < 0.01. Data acquired from 6 independent experiments.

Biofilm dispersal has been linked to swarming motility, which in *P. aeruginosa* depends on pili and flagella^10,60^. Furthermore, the swarming of PAO1 increases upon exposure to the biofilm dispersal signal NO^61^, suggesting a potential link between NO-driven swarming and dispersal. Indeed, the extent to which swarming is inhibited in Δ*rbdA* and Δ*dipA* is consistent with their swimming behaviours, as shown in Figs. 6 and 7. Similarly, the swimming deficient mutant Δ*fliM* also displayed inhibited swarming.

This contrasts with Δ*fimX*, which displays inhibited twitching, but presented a much larger swarming area, that increased further upon NO treatment (119 ± 31% increase). It is worth noting that Δ*fimX* shows a swarming behaviour distinct from other mutants and WT (smooth edges without tendrils). This distinct swarming behaviour is also observed for Δ*pilA*, as is a similar effect on swarming zone after NO treatment (129 ± 33% increase) (Fig. 7a). This observation is consistent with previous reports showing that pili contribute to swarming patterns^60^.

When treated with 1 μM SNP, the swarming zone of WT PAO1 significantly increased 83 ± 32% (P < 0.01), however, Δ*rbdA*, Δ*pa2072* and Δ*dipA* only show 23 ± 13%, 12 ± 8% and 37 ± 24% increases (P < 0.05) respectively, demonstrating impaired swarming responses to NO in these three strains. Similarly, Δ*fliM* and Δ*pilA*Δ*fliM* mutants showed only 35 ± 12% and 26 ± 11% swarming increases upon NO treatment. The variance in the swarming responses upon NO treatment correlates well with different biofilm dispersal responses: NO failed to drive Δ*rbdA*, Δ*pa2072*, Δ*dipA*, Δ*fliM* and Δ*pilA*Δ*fliM* swarming to the same extent as WT, and equally failed to disperse biofilms of these mutants (Figs. 2 and S1). Hence our data suggest a close link between NO-induced swarming ability and biofilm dispersal.

## Discussion

In this study we document how four PAS domain-containing diguanylate cyclases/phosphodiesterases modulate motility behaviours, biofilm phenotypes and dispersal responses. Using deletion mutants, we show that Δ*rbdA* and Δ*pa2072* have almost opposing physiological roles, despite homologous protein domain architectures. The pseudo-enzymes Δ*fimX* and Δ*dipA* regulate the functions of pili and flagellum respectively, modulating motility and presenting opposite effects on NO-induced biofilm dispersal that extend over the simple regulation of c-di-GMP concentration. All four mutants show an altered response to NO-induced biofilm dispersal.

The ability of cyclase and phosphodiesterase enzymes to control specific bacterial phenotypes without cross-talk between different c-di-GMP-responding systems is a feature of much current research and has previously been summarised by Dahlstrom and O’Toole^62^. Our study has mapped the outputs of a series cyclase/phosphodiesterases and suggests that these proteins are working in different pathways to control a range of phenotypes including motility and NO-mediated dispersal responses. The data suggest that the NO signal can have a differential readout, operating through different regulators that impact on motility and dispersal, and that unravelling these pathways may provide a helpful model system with which to understand specificity in c-di-GMP signalling.

In *P. aeruginosa*, bacterial biofilm formation and dispersal are regulated by intracellular c-di-GMP concentrations in response to environmental signals^4,22^. The understanding of enzymes that can regulate c-di-GMP levels, such as diguanylate cyclases (DGCs) and phosphodiesterases (PDEs), and their linked sensor domains will be an essential step to disentangle the network that controls this physiological switch. Bacteria carry these enzymatic activities in multi-domain proteins, allowing for a tight control of catalytic activity. Regulatory domains in these multi-domain proteins may therefore allow functional diversification of these enzymes.

Of the cohort of 41 DGC/PDE proteins in *P. aeruginosa* PAO1, twelve have regulatory PAS domains, as shown in Fig. 1. PAS domains are known as dimerisation domains that respond to a variety of environmental triggers^63,64^. Since DGC and PDE enzymes must dimerise to become catalytically active^3,29,38,65,66^, PAS domains could link sensation of environmental stimuli to dimerisation, and thus provide an elegant solution to integrate signalling with enzyme activation. To investigate the function of these proteins we screened gene deletion mutants for altered biofilm dispersal in response to NO (Fig. 2).

RbdA and PA2072 share an identical domain structure and both respond to the same environmental input, as NO-induced dispersal is inhibited in knock-out strains of both genes, and they present an interesting case for a functional divergence related to output rather than input. We show that both Δ*rbdA* and Δ*pa2072* are different to WT PAO1 in microcolony formation. The five-fold increase of microcolony size in Δ*rbdA* leads to thicker biofilms and is reflected in a two-fold increase in biomass with enhanced total protein and polysaccharide in the EPS. In stark contrast, microcolony sizes are greatly reduced for Δ*pa2072*, with consequent formation of much thinner biofilms than WT PAO1 that reached only one fifth of the biomass after 48 hrs. The phenotypes of Δ*rbdA* and Δ*pa2072* also affected biofilm surface coverage (Fig. 3). Observed phenotypes correlate with c-di-GMP levels, which are higher for Δ*rbdA* but lower for Δ*pa2072*, Fig. 4. The data lead to the surprising conclusion that these homologous proteins perform opposite roles, rather than being redundant.

Several of the 41 *P. aeruginosa* DGC and PDE proteins show amino acid mutations within their catalytic motifs, rendering them catalytically inactive and converting them to pseudo-enzymes, and we identified two such examples in the set of twelve PAS containing DGC/PDE proteins. Our *in silico* analysis and earlier data indicate that FimX is an inactive DGC and has degenerate catalytic EAL and DDFGTG motifs, which would lead to disruption of metal binding at the active site that would impair or abolish PDE activity^38,50,52^. The protein DipA also contains an inactive DGC, but was shown to possess an active PDE domain^43^ (Fig. 1). While *ΔfimX* shows comparable c-di-GMP levels to PAO1 WT, deletion of *dipA* leads to increased levels of intracellular c-di-GMP, suggesting DipA is an active phosphodiesterase *in vivo*^13^, while FimX is not (Fig. 4).

Flagellum mediated swimming motility is significantly reduced in Δ*dipA*, as reported previously^13,67^, while Δ*fimX* shows a twitching defect pointing to altered pili function^68^. It is known that when intracellular c-di-GMP levels are low, the high-affinity of FimX for c-di-GMP makes it possible to interact with PilB and facilitates its localisation, which eventually powers type-IV pili assembly^69^; a requirement that can be bypassed when c-di-GMP levels increase^70^. Furthermore, FimX was shown to connect environmental signals to twitching motility^68^. Similarly, the chemotaxis machinery histidine kinase CheA is required for both polar localization and activity of DipA, leading to heterogeneity in c-di-GMP levels, which in turn controls flagellar-based motility^13^. Our observations in this study therefore lead to two possible suggestions: (1) In response to NO *P. aeruginosa* intracellular c-di-GMP levels are altered, which then affects the subcellular concentrations or conformations of FimX and DipA, with a consequent effect on regulation of pili or flagella, respectively. (2) NO directly modulates the conformation of FimX and DipA through their upstream sensor domains, resulting in altered c-di-GMP concentrations in the cell pole where flagellum and pili reside. Both methods lead to altered bacteria motility and potentially biofilm dispersal.

All four mutants highlight a correlation between swarming motility and NO-triggered biofilm dispersal. Biofilms of Δ*rbdA*, Δ*pa2072* and Δ*dipA* dispersed less than WT upon NO challenge, and accordingly, NO did not affect their swarming motilities to the same extent as WT PAO1. In contrast, enhanced swarming and increased biofilm dispersal were observed for Δ*fimX*. Another example of this behaviour has been provided by previous observations of nitrite reductase (*nirS*) deletion mutants unable to produce NO endogenously, which showed deficiencies in swarming motility and biofilm dispersal^61,71^. This suggests a potential regulatory circuit linking NO, c-di-GMP, swarming behaviour and biofilm dispersal, within which the four proteins studied here may play important roles.

In conclusion, this study highlights the complexity of how bacteria integrate multiple signals from c-di-GMP outputs and provides information on how closely related enzymes involved in c-di-GMP turnover can have opposing effects on biofilm development.

## Supplementary information

Supplementary material.
