# Supplementary material for: Differential impact on motility and biofilm dispersal of closely related phosphodiesterases in Pseudomonas aeruginosa
Source: Sci Rep. 2020 Apr 10;10:6232. doi: 10.1038/s41598-020-63008-5 (PMC7148300; doi:10.1038/s41598-020-63008-5)
Supplement: Supplementary file 1 — Supplementary material. [file 41598_2020_63008_MOESM1_ESM.pdf]

Supplement to:

Differential impact on motility and biofilm dispersal of closely related phosphodiesterases in *Pseudomonas aeruginosa*

Yu-ming Cai[a,b], Andrew Hutchin[b,c,*], Jack Craddock[a,b], Martin A. Walsh[c,d], Jeremy S. Webb[a,b] and Ivo Tews[a,b]

[a] National Biofilms Innovation Centre, University of Southampton, Southampton, SO17 1BJ, UK

[b] Biological Sciences, Institute for Life Sciences, University of Southampton, Southampton, SO17 1BJ, UK

[c] Diamond Light Source, Harwell Science and Innovation Campus, Didcot, Oxfordshire, OX11 0DE, UK

[d] Research Complex at Harwell, Harwell Science and Innovation Campus, Didcot, Oxfordshire, OX11 0FA, UK

[*] Present Address: Structure and Function of Biological Membranes Lab, Université Libre de Bruxelles, Boulevard du Triomphe, 1050 Bruxelles, Belgium

Address correspondence to Ivo Tews, ivo.tews@soton.ac.uk

JSW and IT are last authors on this work

Table S1: Bacterial strains and plasmids

**Strains and plasmids**	Genotype or phenotypes[a]	Reference
E.coli		
S17-1(λpir)	*TpR SmR recA, thi, pro, hsdR-M+RP4*: 2-Tc:Mu: Km Tn7	Biomedal corp.
DH5α	*spuE44* Δ*lacU169*(ϕ80*lacZ*ΔM15) *hsdR17* λpir *recA1 endA1 gyrA96 thi-1 relA1*	Lab collection
P. aeruginosa		
PAO1	Wild-Type, C.Manoil lab, University of Washington	[57]
**KO mutants**		
Δ*pa0285*	PAO1 mutant (*pa0285::aacCl*); Gm^R	This study
Δ*pa0290*	PAO1 mutant (*pa0290::aacCl*); Gm^R	This study
Δ*pa0338*	PAO1 mutant (*pa0338::aacCl*); Gm^R	This study
Δ*pa0575*	PAO1 mutant (*pa0575::aacCl*); Gm^R	This study
Δ*pa0847*	PAO1 mutant (*pa0847::aacCl*); Gm^R	This study
Δ*pa0861 (*Δ*rbdA)*	PAO1 mutant (*pa0861::aacCl*); Gm^R	This study
Δ*pa1181 (*Δ*yegE)*	PAO1 mutant (*pa1181::aacCl*); Gm^R	This study
Δ*pa2072*	PAO1 mutant (*pa2072::aacCl*); Gm^R	This study
Δ*pa4601 (*Δ*morA)*	PAO1 mutant (*pa4601::aacCl*); Gm^R	This study
Δ*pa4959 (*Δ*fimX)*	PAO1 mutant (*pa4959::aacCl*); Gm^R	This study
Δ*pa5017 (*Δ*dipA)*	PAO1 mutant (*pa5017::aacCl*); Gm^R	This study
Δ*pa5442*	PAO1 mutant (*pa5442::aacCl*); Gm^R	This study
Δ*pa1443 (*Δ*fliM)*	PAO1 mutant (*pa4959::aacCl*); Gm^R	This study
Δ*pa4525 (*Δ*pilA)*	PAO1 mutant (*pa5017::aacCl*); Gm^R	This study
Δ*pa1443*Δ*pa4525 (*Δ*pilA*Δ*fliM)*	PAO1 mutant (*pa1443::aacCl,pa4525::Neo*); Gm^R; Km^R	This study
**KO mutants with c-di-GMP reporter gfp vector**		
PAO1 Tn7CdrA::*gfp*	PAO1 WT with mini-Tn7-P_{cdrA}-RBSII-*gfp*(Mut3)-T_0-T_1;Tet^R	This study
Δ*pa0285* Tn7CdrA::*gfp*	PAO1 mutant (*pa0285::aacCl*) with mini-Tn7-P_{cdrA}-RBSII-*gfp*(Mut3)-T_0-T_1;Gm^R;Tet^R	This study
Δ*pa0290* Tn7CdrA::*gfp*	PAO1 mutant (*pa0290::aacCl*) with mini-Tn7-P_{cdrA}-RBSII-*gfp*(Mut3)-T_0-T_1;Gm^R;Tet^R	This study
Δ*pa0338* Tn7CdrA::*gfp*	PAO1 mutant (*pa0338::aacCl*) with mini-Tn7-P_{cdrA}-RBSII-*gfp*(Mut3)-T_0-T_1;Gm^R;Tet^R	This study
Δ*pa0575* Tn7CdrA::*gfp*	PAO1 mutant (*pa0575::aacCl*) with mini-Tn7-P_{cdrA}-RBSII-*gfp*(Mut3)-T_0-T_1;Gm^R;Tet^R	This study
Δ*pa0847* Tn7CdrA::*gfp*	PAO1 mutant (*pa0847::aacCl*) with mini-Tn7-P_{cdrA}-RBSII-*gfp*(Mut3)-T_0-T_1;Gm^R;Tet^R	This study
Δ*pa0861* Tn7CdrA::*gfp*	PAO1 mutant (*pa0861::aacCl*) with mini-Tn7-P_{cdrA}-RBSII-*gfp*(Mut3)-T_0-T_1;Gm^R;Tet^R	This study
Δ*pa1181* Tn7CdrA::*gfp*	PAO1 mutant (*pa1181::aacCl*) with mini-Tn7-P_{cdrA}-RBSII-*gfp*(Mut3)-T_0-T_1;Gm^R;Tet^R	This study
Δ*pa2072* Tn7CdrA::*gfp*	PAO1 mutant (*pa2072::aacCl*) with mini-Tn7-P_{cdrA}-RBSII-*gfp*(Mut3)-T_0-T_1;Gm^R;Tet^R	This study
Δ*pa4601* Tn7CdrA::*gfp*	PAO1 mutant (*pa4601::aacCl*) with mini-Tn7-P_{cdrA}-RBSII-*gfp*(Mut3)-T_0-T_1;Gm^R;Tet^R	This study
Δ*pa4959* Tn7CdrA::*gfp*	PAO1 mutant (*pa4959::aacCl*) with mini-Tn7-P_{cdrA}-RBSII-*gfp*(Mut3)-T_0-T_1;Gm^R;Tet^R	This study
Δ*pa5017* Tn7CdrA::*gfp*	PAO1 mutant (*pa5017::aacCl*) with mini-Tn7-P_{cdrA}-RBSII-*gfp*(Mut3)-T_0-T_1;Gm^R;Tet^R	This study
Δ*pa5442* Tn7CdrA::*gfp*	PAO1 mutant (*pa5442::aacCl*) with mini-Tn7-P_{cdrA}-RBSII-*gfp*(Mut3)-T_0-T_1;Gm^R;Tet^R	This study
**Plasmids**		
pPS856	Ap^R, Gm^R; 0.83-kb blunt-ended SacI fragment from pUCGM	[37]
pEX100T	Ap^R; $oriT^+$ $sacB^+$ gene replacement vector	[37]
pCdrA::*gfp*	pUCP22Not-P_{cdrA}-RBSII-*gfp*(Mut3)-T_0-T_1, Amp^R Tet^R	[41]
pCR™4-TOPO™	Km^R and Amp^R TOPO cloning expression vector	Invitrogen™

Table S2: Primer sequences

Primer	Sequence (5'→3')
**Used for KO mutant generation and sequencing**	
Δpa0285	
PA0285-up-F	ATGC*CCCGGG* CCTGACCGACAATCTCGAC
PA0285-up-R	ATGC*GAATTC* GTTCCAGATCTCTCCGCTC
PA0285-dn-F	ATGC*AAGCTT* AGTCACCGAAAGCGCGG
PA0285-dn-R	ATGC*CCCGGG* TCAGTCTTCCGGCAGCG
SeqPA0285-F	GTAACTGCCACCAGTTGC
SeqPA0285-R	CCATCCAGGCTTCTGAG
Δpa0290	
PA0290-up-F	ATGC*CCCGGG* GCGAATGGTCTACGTCAGC
PA0290-up-R	ATGC*GAATTC* CAGGAACGCCAACGGTATC
PA0290-dn-F	ATGC*AAGCTT* GCGACCTGTTCGGGC
PA0290-dn-R	ATGC*CCCGGG* TCAGCCCACGACGATG
SeqPA0290-F	GTATCGCTCGGCAACTG
SeqPA0290-R	CCTTTCTGGCGAGTTACC
Δpa0338	
PA0338-up-F	ATGC*CCCGGG* GTGCGGCGCTTACGC
PA0338-up-R	ATGC*GAATTC* CTCGGGATGGATGATGC
PA0338-dn-F	ATGC*AAGCTT* CCTGTTCGGCCACTCC
PA0338-dn-R	ATGC*CCCGGG* TCAGCAACAGGCCACG
SeqPA0338-F	CAAGGTGAAGTGGCTGC
SeqPA0338-R	CTCCAGCGTGTTCATCG
Δpa0575	
PA0575-up-F	ATGC*CCCGGG* GATCATCATCCTGGCACG
PA0575-up-R	ATGC*GAATTC* GGACGATGGGAAGACCG
PA0575-dn-F	ATGC*AAGCTT* GCCGAGGATTGCGG
PA0575-dn-R	ATGC*CCCGGG* CACCGTGAGCTGCATG
SeqPA0575-F	GACGAGTGACGAACGAAC
SeqPA0575-R	GTTGCCATCAGATAAGGAC
Δpa0847	
PA0847-up-F	ATGC*CCCGGG* CCGTCGCCGAGGAC
PA0847-up-R	ATGC*GAATTC* GTCGTAGCTGGCATCCC
PA0847-dn-F	ATGC*AAGCTT* CGCGCCATGCAGAG
PA0847-dn-R	ATGC*CCCGGG* CGCCCAGGCGGTAG
SeqPA0847-F	GGAACTTATCCATGCGGTTG
SeqPA0847-R	GTGGCTGAACTGCTGCATC
Δpa0861	
PA0861-up-F	ATGC*CCCGGG* ATGAGGCAGAACCGGACTC
PA0861-up-R	ATGC*GAATTC* AGCTCGATGGCGCG
PA0861-dn-F	ATGC*AAGCTT* GGTTGCCAACCTGGCC
PA0861-dn-R	ATGC*CCCGGG* CTACCGGAGGTTCTGTCCC
SeqPA0861-F	CAGCCACTAGACTCCTACTG
SeqPA0861-R	CAGTCGACGATCAGTTGC
Δpa1181	
PA1181-up-F	ATGC*CCCGGG* TTCGCGTGCTCTGGG
PA1181-up-R	ATGC*GAATTC* AGGCCATCGGCAGGTAC
PA1181-dn-F	ATGC*AAGCTT* CTTCGAGCTGACCGAGACG
PA1181-dn-R	ATGC*CCCGGG* TCAGCCCAACTCCTGGC
SeqPA1181-F	CTGAGCCTGGACGATTC
SeqPA1181-R	CTGCACATGAGTTCGCAG

Δ*pa2072*	
PA2072-up-F	ATGC*CCCGGG* ATGCTTGCTTCAGCGATTG
PA2072-up-R	ATGC*GAATTC* TCGACGAAGACCATCACC
PA2072-dn-F	ATGC*AAGCTT* AGGCGAGGTGATGGTCTC
PA2072-dn-R	ATGC*CCCGGG* TGAGCAGGAACCCTTGC
SeqPA2072-F	ATGAAGGTCGAGGTGGTG
SeqPA2072-R	TGACGCCTCGTTGTTATTGC
Δ*pa4601*	
PA4601-up-F	ATGC*CCCGGG*TTCGCGGACTCTACGACAG
PA4601-up-R	ATGC*GAATTC*AACTCGCTACGGGTCTCG
PA4601-dn-F	ATGC*AAGCTT*ATCCTGATGAGTGACGTCGC
PA4601-dn-R	ATGC*CCCGGG*TCAGCCCTCGTTGAACATG
SeqPA4601-F	GACAAAGTGCCTCGAC
SeqPA4601-R	GTCAAATCACGGCTGAAC
Δ*pa4959*	
PA4959-up-F	ATGC*CCCGGG* CACGTACGACGGCGAAC
PA4959-up-R	ATGC*GAATTC* GTTCTCGACTTTCTTCAGCAG
PA4959-dn-F	ATGC*AAGCTT* GAGGTGCTCCTGCGC
PA4959-dn-R	ATGC*CCCGGG* GGTCCTGGACGAAGGAG
SeqPA4959-F	GTTCGAATACCGCTCG
SeqPA4959-R	TGGACGGCATCCTCTAC
Δ*pa5017*	
PA5017-up-F	ATGC*CCCGGG* CGACTACCCTCTGCGC
PA5017-up-R	ATGC*GAATTC* GTGCCGTAGGCTTCGC
PA5017-dn-F	ATGC*AAGCTT* GCCGATCGGCAAGTG
PA5017-dn-R	ATGC*CCCGGG* CCATGGCGATCACCG
SeqPA5017-F	CAATCGGCATAATGCACC
SeqPA5017-R	CAGAACCATGGCTCTCG
Δ*pa5442*	
PA5442-up-F	ATGC*CCCGGG* ATGACCGTCCATGTCGAG
PA5442-up-R	ATGC*GAATTC* CGAACACCAGCACCTGC
PA5442-dn-F	ATGC*AAGCTT* CGGCGCAACTGGAGC
PA5442-dn-R	ATGC*CCCGGG* TCAACGAGCCTGGCG
SeqPA5442-F	GTCGTTGAGGGAGTTCAG
SeqPA5442-R	GACCAATCGGGATAGCAG
Δ*pa1443*	
PA1443-up-F	ATGC*CCCGGG* AGCAAGCTGAAGCTGATC
PA1443-up-R	ATGC*GAATTC* TTTCATCCTGGGAAAGCAG
PA1443-dn-F	ATGC*AAGCTT* TACAGATTCTCGAAGCGG
PA1443-dn-R	ATGC*CCCGGG* AGCTTCTTGATGCGTTCG
SeqPA1443-F	AGCAAGCTGAAGCTGATC
SeqPA1443-R	AGCTTCTTGATGCGTTCG
Δ*pa4525*	
PA4525-up-F	ATGC*CCCGGG* TTGTCCAGGCTATTCAGG
PA4525-up-R	ATGC*GAATTC* ACCACGATCATCAGTTCGATC
PA4525-dn-F	ATGC*AAGCTT* TACCTTTACCTTCCAGACTG
PA4525-dn-R	ATGC*CCCGGG* TGACCAAGGATGTCAGG
SeqPA4525-F	TTGTCCAGGCTATTCAGG
SeqPA4525-R	TGACCAAGGATGTCAGG
Gentamicin resistance and kanamycin resistance gene cassettes	
Gm-F	ATGC*GAATTC* GAATTGACATAAGCCTGTTCGG
Gm-R	ATGC*AAGCTT* GAATTGGCCGCGG
Km-F	ATGC*GAATTC* AATGTCAGCTACTGGGCTATC
Km-R	ATGC*AAGCTT* CTGATGCGGTGTGAAATAC

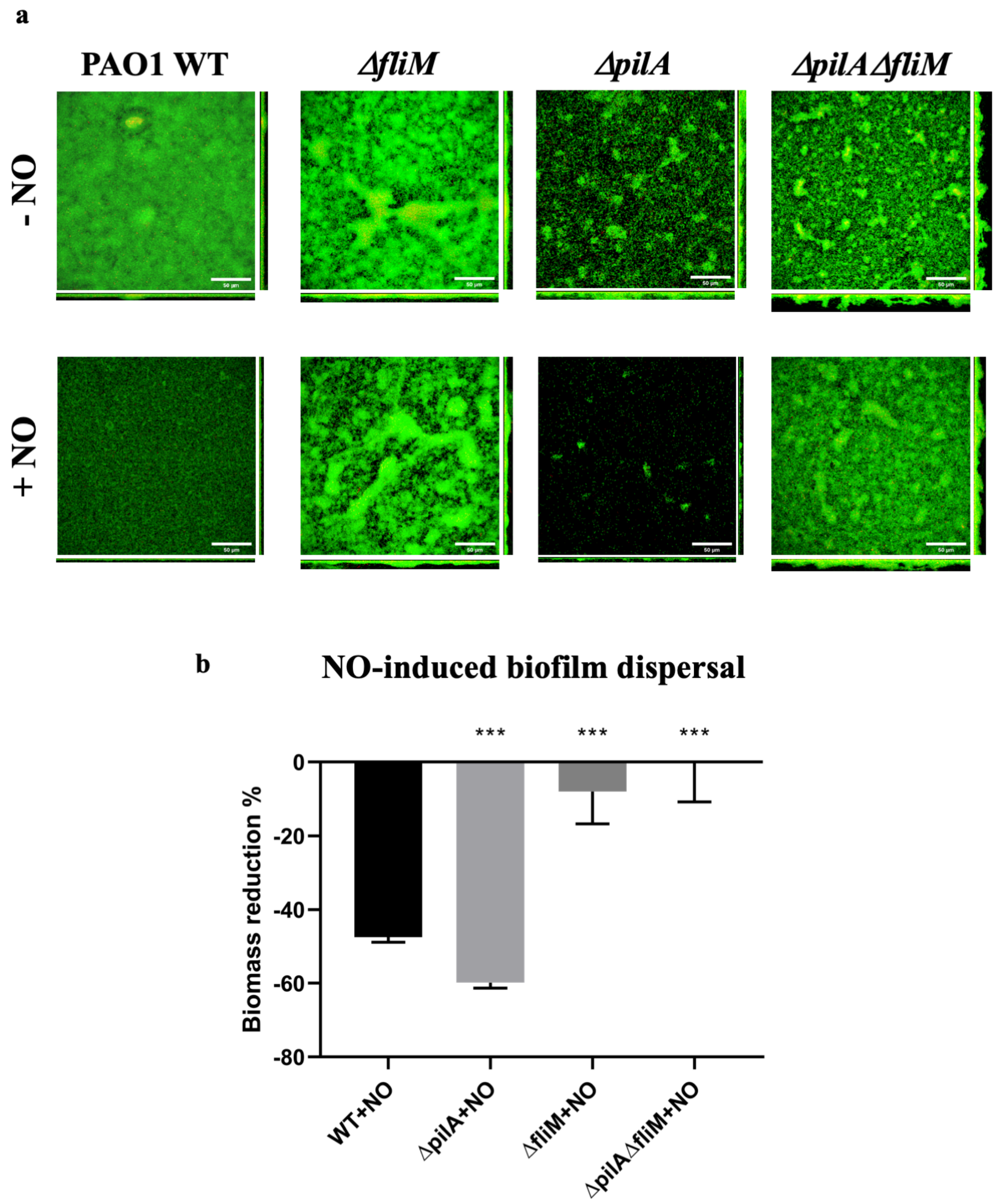

Figure S1. NO-induced biofilm dispersal of flagellum and pili deletion mutants. (A) Confocal laser scanning microscopy images used for phenotypic analysis of 72 hrs mature biofilms before or after 2 hrs treatment with 250 µM SNP for the flagellum mutant Δ*fliM,* the pili mutant Δ*pilA* and the double mutant Δ*pilA*Δ*fliM* compared with PAO1 WT (scale bar 50 µm). **(B)** COMSTAT analysis of biomass reduction after treatment normalised against WT, highlighting differences in biomass reduction for the mutants. The Student's T-test was used to determine significances, where *** denotes a confidence level of $p<0.01$, and ** denotes a confidence level of $0.01<p<0.05$. Data acquired from 3 independent experiments.
